# Beyond western paradigms: a cross-cultural review of social–emotional competence frameworks and the promise of AI-driven assessment

**DOI:** 10.3389/fpsyg.2026.1763245

**Published:** 2026-04-17

**Authors:** Yang Liu, Jiayi Ma, Yin Huang

**Affiliations:** Xinjiang Key Laboratory of Mental Development and Learning Science, College of Psychology, Xinjiang Normal University, Urumqi, China

**Keywords:** artificial intelligence, cross-cultural assessment, framework development, measurement invariance, social–emotional competence, systematic review

## Abstract

**Background:**

Social–emotional competence (SEC) is a critical yet culturally embedded construct. While foundational frameworks like collaborative for academic, social, and emotional learning (CASEL) and OECD provide influential models, their cross-cultural applicability and the validity of associated assessments are increasingly questioned, particularly in non-Western contexts. Concurrently, artificial intelligence (AI) presents novel opportunities for culturally responsive SEC measurement.

**Objective:**

This systematic review synthesizes literature on the conceptualizations, theoretical frameworks, and measurement tools for adolescent SEC across diverse cultural settings, with a specific focus on the role of emerging digital technologies.

**Methods:**

Following PRISMA 2020 guidelines, a comprehensive search of Web of Science and Google Scholar (2000–2025) was conducted. After AI-assisted triage and manual screening, 89 studies were included. A narrative synthesis was performed, employing thematic analysis to compare frameworks (e.g., CASEL, OECD, Chinese, Australian/New Zealand models) across theoretical foundations, operational dimensions, and measurement approaches.

**Results:**

The analysis reveals that dominant SEC frameworks are cultural artifacts reflecting underlying individualist or collectivist values, leading to divergent prioritizations of competencies (e.g., autonomy vs. harmony). Achieving cross-cultural measurement invariance for standardized tools remains a significant challenge, necessitating strategies like anchoring vignettes and emic–etic integration. The review identifies a clear trajectory toward technology-enhanced assessment, highlighting the potential of multimodal AI analysis, generative AI for stimuli creation, virtual reality simulations, and large language models to enable more ecologically valid, behavioral, and culturally configurable evaluations. However, these technologies introduce risks of algorithmic bias and digital colonialism.

**Conclusion:**

Advancing the field requires a pluralistic, dialogical approach that decentralizes Western models, invests in indigenous theory-building, and ethically harnesses technology. Future research must develop assessment methodologies that balance generalizability with deep cultural respect, leveraging AI as a tool for empowerment and context-rich insight rather than for imposing reductionist, cross-cultural rankings.

## 1 Introduction

Social–emotional education is an integral part of the education system, aimed at nurturing adolescents' emotional literacy, interpersonal skills, and positive emotional attitudes and values. The efficacy of social–emotional education closely aligns with Goals 3 and 4 of the Sustainable Development Goals (SDGs; Cefai et al., 2018; García-Gil et al., 2023). This educational endeavor is conceptualized through two interrelated constructs: social–emotional competence (SEC) and social–emotional learning (SEL). SEC represents the holistic set of cognitive, affective, and behavioral capabilities that enable individuals to understand and manage their own emotions, establish positive relationships, make responsible decisions, and interact effectively within social contexts (Collie, 2020; Schoon, 2021). It is the measurable outcome or skill set encompassing core domains such as self-awareness, self-management, social awareness, relationship skills, and responsible decision-making. Conversely, SEL refers to the process through which these competencies are developed—the systematic, evidence-based educational approaches, programs, and practices designed to foster SEC in learners (Al-Hroub and Al-Hroub, 2024). Thus, social–emotional education endorses the cultivation of adolescents' SEC through SEL, which not only encourages their personal sustainable development but also stimulates societal sustainability (Arbués et al., 2025).

At the individual level, higher SEC contributes to enhanced psychological wellbeing and prepares adolescents for future life challenges. At the societal level, widespread high social–emotional competence among adolescents fosters robust social responsibility and civic cognizance, nurturing a more harmonious and inclusive society.

Current research indicates that Western academia has developed a relatively mature conceptual system, theoretical framework, and assessment tools for SEC, rooted in an individualistic cultural context that emphasizes personal autonomy, emotional expression, and self-actualization (Collie et al., 2024). In contrast, East Asian collectivist cultures, represented by China, Japan, and South Korea, prioritize interpersonal harmony, emotional restraint, responsibility, and collective benefit. Empirical studies have revealed both commonalities and differences in core perceptions of SEC across these cultural groups (Lee and Junus, 2024).

Existing research has largely focused on SEC within single cultural contexts, while cross-cultural comparative studies remain fragmented. There is a lack of systematic cultural comparison across three core dimensions: conceptualization, framework development, and assessment tools. This gap has led to a “poor fit” of well-established Western theories and instruments in non-Western cultural contexts such as East Asia, resulting in distorted assessment outcomes and ineffective cultivation practices (Abd Hadi et al., 2023). Concurrently, the integration of digital and artificial intelligence (AI) technologies is increasingly recognized as a promising avenue for assessing SEC across diverse cultural settings (Belay et al., 2025). To address this, the present paper adopts a cross-cultural comparative perspective, synthesizes relevant literature, and systematically examines differences in the conceptualization logic, framework construction, and cultural adaptability of assessment tools for adolescents' SEC. By clarifying the profound influence of cultural context on research in this field, this study aims to address the fragmentation in existing scholarship and lay a theoretical foundation for cross-cultural dialogue and localized research.

This systematic review responds to the aforementioned needs by conducting a comprehensive synthesis of identified literature, gathered through a rigorous and transparent search of major academic databases. Its primary objectives are threefold: (1) to detail and critically appraise the predominant research methodologies employed in contemporary cross-cultural SEC research; (2) to provide an integrated, thematic synthesis of evidence pertaining to the conceptualizations, frameworks (or core dimensions), and measurement tools of SEC across different cultural contexts; and (3) to explore the emerging digital and analytical frontier by examining how technology and data science are transforming the assessment of SEC in cross-cultural research, thereby outlining a foundation for future innovation. By offering this consolidated and critical overview, the review aims to inform researchers, practitioners, and policymakers, and to chart a course for future inquiry dedicated to enhancing the social–emotional well-being of children and adolescents globally.

## 2 Methods

This systematic review was conducted and reported in accordance with the Preferred Reporting Items for Systematic Reviews and Meta-Analyses (PRISMA) 2020 guidelines. A pre-specified protocol was established to ensure a transparent and reproducible process for identifying, selecting, and synthesizing literature on the theories, frameworks, and measurement of social and emotional competence (SEC).

### 2.1 Eligibility criteria

Studies were included based on the following pre-defined criteria:

**Population:** The study sample primarily consisted of adolescents (typically defined as individuals aged 6–19 years). Studies focusing on children, young adults, or mixed-age populations were included only if they reported adolescent-specific data or the findings were explicitly generalizable to the adolescent population. Studies focused exclusively on other age groups (e.g., early childhood, adulthood) were excluded.

**Concept:** The primary focus of the study had to be on the theory, conceptual framework, model, assessment, or measurement of social and emotional competence, skills, or learning SEC. This included:

- Studies proposing, testing, or comparing theoretical models of SEC.
- Studies developing, validating, or critically reviewing measurement instruments (e.g., questionnaires, scales, observational tools).
- Systematic reviews or meta-analyses focused on SEC theories or measurement.

**Context:** Any setting or context was eligible.

**Study design:** Original empirical research articles (e.g., instrument validation studies, factor analyses, cross-sectional or longitudinal studies testing theoretical models), systematic reviews, scoping reviews, and theoretical papers were included. Editorials, commentaries, and study protocols without results were excluded.

**Publication type and language:** Peer-reviewed journal articles (ARTICLE) and reviews (REVIEW) published in English were included. Gray literature, conference abstracts, and book chapters were excluded.

**Timeframe:** The search covered publications from January 1, 1995, to December 31, 2025, to capture the foundational and evolving discourse in the field.

### 2.2 Search strategy

A comprehensive literature search was conducted across two major academic platforms to ensure broad coverage and mitigate database bias.

**Web of science (WoS) core collection:** The search was performed across the science citation index expanded (SCI-EXPANDED), social sciences citation index (SSCI), arts and humanities citation index (AHCI), and emerging sources citation index (ESCI). The search strategy used a topic search (TS) with Boolean operators and wildcards to account for variations in terminology:

TS = (((social and emotional OR social-emotional OR socioemotional) AND (competenc^*^ OR skill^*^ OR abilit^*^ OR learning OR SEL)) AND (theor^*^ OR framework^*^ OR model^*^ OR conceptual^*^ OR assess^*^ OR measure^*^ OR evaluat^*^ OR instrument^*^ OR questionnaire^*^ OR scale^*^ OR psychometric^*^ OR valid^*^) AND (adolescen^*^ OR teen^*^ OR youth OR “young people”))

**Google scholar:** To capture publications not indexed in WoS and to apply a more flexible, semantic search, a parallel search was conducted using the Google Scholar interface for Google Scholar. The search employed precise phrase matching and combined key conceptual clusters:

- **Core construct:** “Social and Emotional Competence” OR “social emotional competence.”
- **Theory/framework:** “Social and Emotional Competence framework” OR “CASEL framework” OR “social emotional learning theory.”
- **Measurement:** “Social and Emotional Competence measurement” OR “SEC-Q” OR “SELS” OR “social emotional competence assessment.”

Searches on both platforms were conducted in February 2026. All retrieved records were exported to citation management software (EndNote), where duplicates were removed.

### 2.3 Study selection process

**Figure 1:**
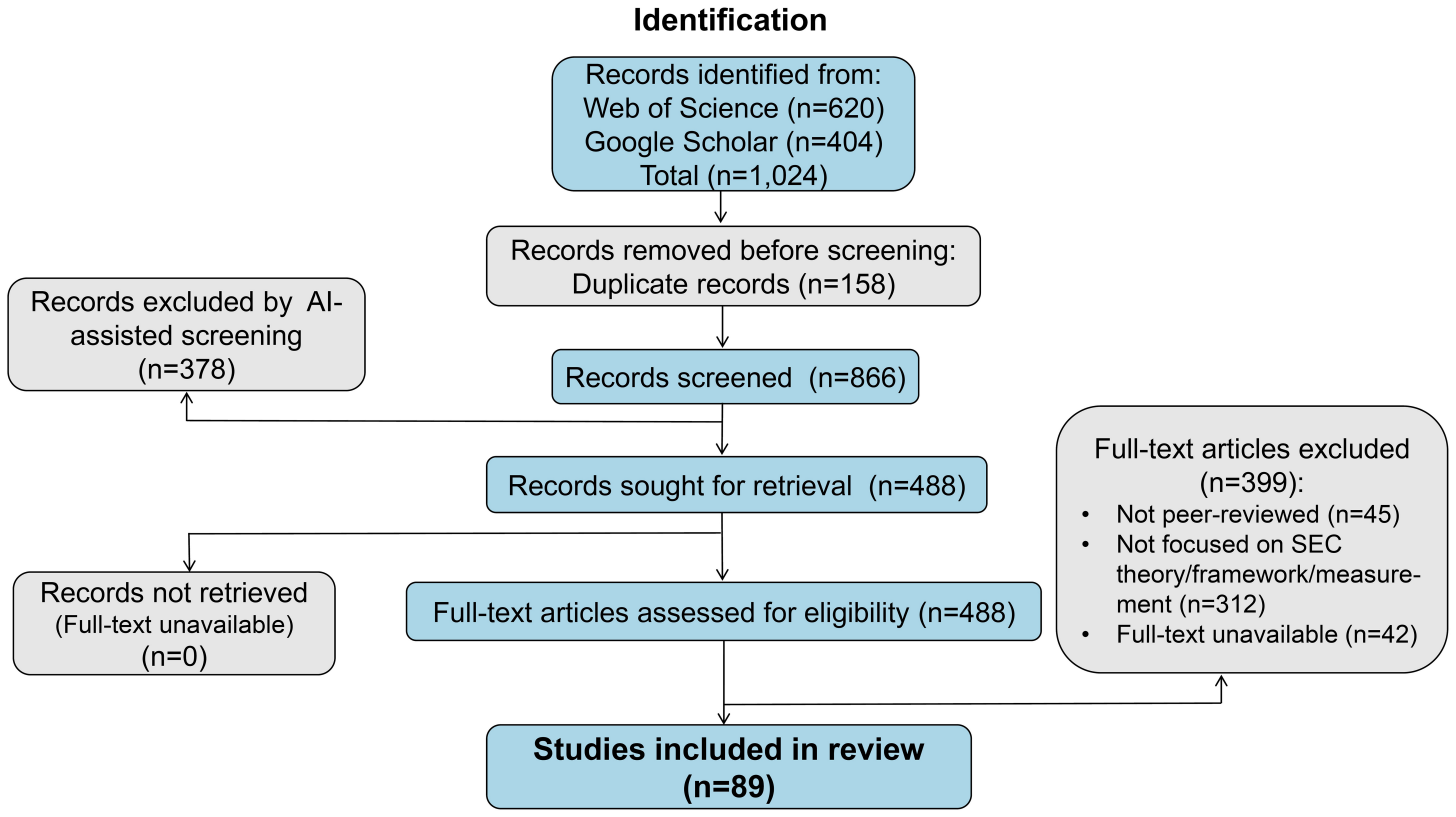
PRISMA 2020 flow diagram of the study selection process. This diagram details the identification, screening, eligibility assessment, and inclusion of studies for the systematic review. AI, artificial intelligence; SEC, social–emotional competence. Source: Authors' work.

The selection process followed the PRISMA 2020 flow diagram (see Figure 1).

- **Identification:** Initial searches yielded 1,024 records (620 from WoS, 404 from Google Scholar). After removing 158 duplicates, 866 unique records remained for screening.
- **AI-assisted title/abstract screening:** To manage the volume, an initial triage was performed using a large language model (LLM). The LLM was prompted to classify each record as “Include,” “Exclude,” or “Unclear” based on the eligibility criteria, with a conservative bias toward inclusion for any record potentially related to SEC theory or measurement.
- **Manual screening:** Two independent reviewers manually screened all records flagged by the AI as “Include” or “Unclear” (*n* = 488), plus a random 10% sample of those flagged as “Exclude” to verify the AI's accuracy. Discrepancies were resolved through discussion or by a third reviewer. This stage resulted in 142 articles selected for full-text retrieval.
- **Full-text assessment:** The full texts of the 142 articles were independently assessed for eligibility by two reviewers. A final set of 89 studies met all inclusion criteria and formed the corpus for this synthesis. The primary reasons for exclusion at this stage were: not peer-reviewed, not focused on SEC theory/framework/measurement (e.g., focused solely on intervention outcomes), or the full text was unavailable.

### 2.4 Data extraction and appraisal of methodological rigor

To ensure a rigorous and transparent synthesis, a two-stage process was implemented following study selection: standardized data extraction and a critical appraisal of the methodological rigor of the included studies.

#### 2.4.1 Data extraction

A standardized data extraction form was developed. One reviewer performed the initial extraction for all 89 included studies, capturing detailed information across five domains. A second reviewer independently verified the accuracy and completeness of the extracted data, with any discrepancies resolved through discussion. The form was designed to capture both the substantive content and methodological characteristics of each study, encompassing:

- **Study identification and context:** Authors, publication year, country, publication title, and stated primary aim of the study (e.g., theory development, instrument validation, intervention evaluation).
- **Conceptual and theoretical focus:** The specific social–emotional competence (SEC) or related construct(s) of primary interest; description of the underlying theoretical model or framework (e.g., CASEL, Big Five, Indigenous wellbeing models).
- **Methodology and sample:** Study design (e.g., randomized controlled trial, cross-sectional survey, psychometric validation, qualitative study), sample size, and detailed participant demographics (e.g., age, cultural background, clinical or educational setting).
- M**easurement and assessment:** Name and description of all primary assessment tools used; detailed reporting of psychometric properties (e.g., reliability coefficients, validity evidence, cross-cultural measurement invariance metrics, where applicable).
- **Key findings and conclusions:** The main empirical results or theoretical conclusions, particularly as they pertained to SEC conceptualization, framework utility, or measurement properties.

#### 2.4.2 Appraisal of methodological rigor and evidence strength

Given the significant heterogeneity in study designs—spanning theoretical papers, psychometric analyses, intervention trials, and large-scale surveys—a unitary, standardized quality assessment tool (e.g., Cochrane ROB-2) was neither feasible nor conceptually appropriate. Instead, we adopted a pragmatic, design-informed, and evidence-graded approach to critically appraise the methodological strength of each study, directly informing the subsequent narrative synthesis (Section 2.5).

**This appraisal involved two key steps during the extraction and review process:** Documentation of design-specific methodological markers: We systematically cataloged indicators of rigor pertinent to each study's design. For psychometric studies, this included noting reported indices of reliability (e.g., Cronbach's alpha), validity (factor structure, convergent/divergent validity), and tests of cross-cultural measurement invariance. For intervention or outcome studies, we recorded critical design features such as the use of control/comparison groups, randomization procedures, sample size justification, attrition rates, and the depth of cultural adaptation described. For large-scale assessments (e.g., OECD SSES), we noted the sophistication of sampling, translation protocols, and methods for ensuring cross-cultural comparability (e.g., use of anchoring vignettes).

**Informing evidence weighting for synthesis:** The methodological profile generated for each study served as a critical lens for the thematic analysis in Section 2.5. This allowed for an evidence-graded synthesis where the contribution of a study's findings to the review's conclusions was consciously weighted. Robust conclusions about, for instance, the factor structure of SEC measures are primarily anchored in studies employing advanced psychometric methods. Claims regarding intervention efficacy rely more heavily on evidence from well-designed RCTs. Insights from exploratory qualitative or theoretical studies are valued for generating nuanced hypotheses and identifying cultural context but are explicitly framed as indicative rather than definitive. This transparent approach ensures the final synthesis acknowledges the spectrum of evidence strength, providing a more nuanced and credible foundation for the conclusions drawn in Sections 3, 4.

### 2.5 Data synthesis

Given the significant heterogeneity in the included study designs, research questions, and cultural contexts, a narrative synthesis approach guided by iterative thematic analysis was employed. This inductive, multi-phase process was designed to move from a close reading of individual studies to the identification of the overarching analytical themes that structure the results section, ensuring the synthesis was firmly grounded in the evidence.

#### 2.5.1 Phase 1: immersion and initial familiarization

Following data extraction, the two reviewers independently immersed themselves in the full dataset of 89 studies, reading and re-reading the extracted information for each. The focus was on understanding the primary aim, conceptual or methodological focus, cultural context, and key findings of each piece of literature. This stage fostered a deep, holistic familiarity with the corpus, moving beyond the extracted data points to grasp the narrative and contribution of each study.

#### 2.5.2 Phase 2: iterative coding and theme development

Building on this immersion, an initial set of descriptive codes was generated through an open coding process. Codes captured the salient features of each study, such as “CASEL framework application,” “Big Five model validation,” “measurement invariance testing,” “Confucian values integration,” “Indigenous wellbeing model,” or “AI-based assessment tool.” Through constant comparison, codes were grouped into preliminary thematic clusters based on conceptual and methodological similarities. For instance, a clear cluster emerged around studies centrally focused on the CASEL framework outside the U.S. (e.g., McKown, 2019; Anziom et al., 2021; Hart et al., 2023). Another distinct cluster comprised literature critically examining the adaptation and application of the OECD's SSES framework and its psychometric properties in cross-cultural settings (e.g., Chernyshenko et al., 2018; Wang and King, 2024). Separate clusters formed around scholarship on SEC conceptualizations within China (e.g., You, 2023; Du and Li, 2024), and frameworks from Australia and Aotearoa New Zealand integrating Indigenous perspectives (e.g., Dudgeon et al., 2025; Sangha et al., 2024). Concurrently, prominent clusters were identified concerning the methodological challenge of cross-cultural measurement invariance (e.g., Paccagnella, 2021; Menold et al., 2023) and the emerging role of digital technologies and AI in assessment (e.g., Indellicato, 2024; Brooks et al., 2024).

#### 2.5.3 Phase 3: theme refinement and structuring of the synthesis

In the final phase, these preliminary clusters were refined, defined, and structured into the coherent analytical framework presented in Section 3. The goal was to ensure themes were mutually exclusive, collectively exhaustive, and logically sequenced. The central comparative analysis of frameworks (Section 3.2) was organized into four sub-themes (OECD, CASEL, Chinese, Australian/New Zealand) based on their distinct geographical-cultural origins, foundational theoretical paradigms, and primary domains of application or policy influence. The themes on measurement invariance (Section 4.2) and AI in assessment (Section 4.3) were elevated as cross-cutting analytical themes because they represent critical, field-wide challenges and frontiers that emerged from the analysis of all framework discussions. This structured, iterative process ensures a transparent audit trail from the raw data of the included studies to the synthesized findings, providing the methodological rigor requested for a systematic review.

## 3 Results

### 3.1 Study selection and characteristics

The final corpus for this review comprised 89 studies. The publication dates spanned from 2000 to 2025, with a marked concentration of studies published in the 2020s, reflecting accelerated growth in the field. The research exhibited a global distribution, with contributions from North and South America, Europe, Asia, Africa, and Australia. Methodologies were diverse: approximately 5.6% were randomized controlled trials (RCTs) or cluster RCTs; 12.4% were longitudinal or prospective cohort studies; 44.9% were cross-sectional surveys; 13.5% were psychometric or validation studies; 16.9% were systematic or scoping reviews; and 6.7% employed mixed-methods designs. Sample sizes ranged from small, in-depth qualitative cohorts (*n* < 10) to large-scale national and international datasets (e.g., OECD Survey on Social and Emotional Skills, Longitudinal Study of Australian Children, CASEL Survey on) with samples exceeding 100,000 participants.

### 3.2 A comparative analysis of major social and emotional competence frameworks

#### 3.2.1 The CASEL framework: a systemic, education-focused model

The collaborative for academic, social, and emotional learning (CASEL) framework is the most influential model driving SEL practice in schools, particularly in the United States, with growing international adoption.

**Theoretical foundation:** CASEL integrates developmental, organizational, and educational psychology (McKown, 2019). It is less tied to a single personality theory and more focused on malleable, teachable competencies that can be developed through deliberate instruction and supportive environments (Hart et al., 2023). It emphasizes a systemic approach involving classrooms, schools, families, and communities (McKown, 2019).

**Figure 2:**
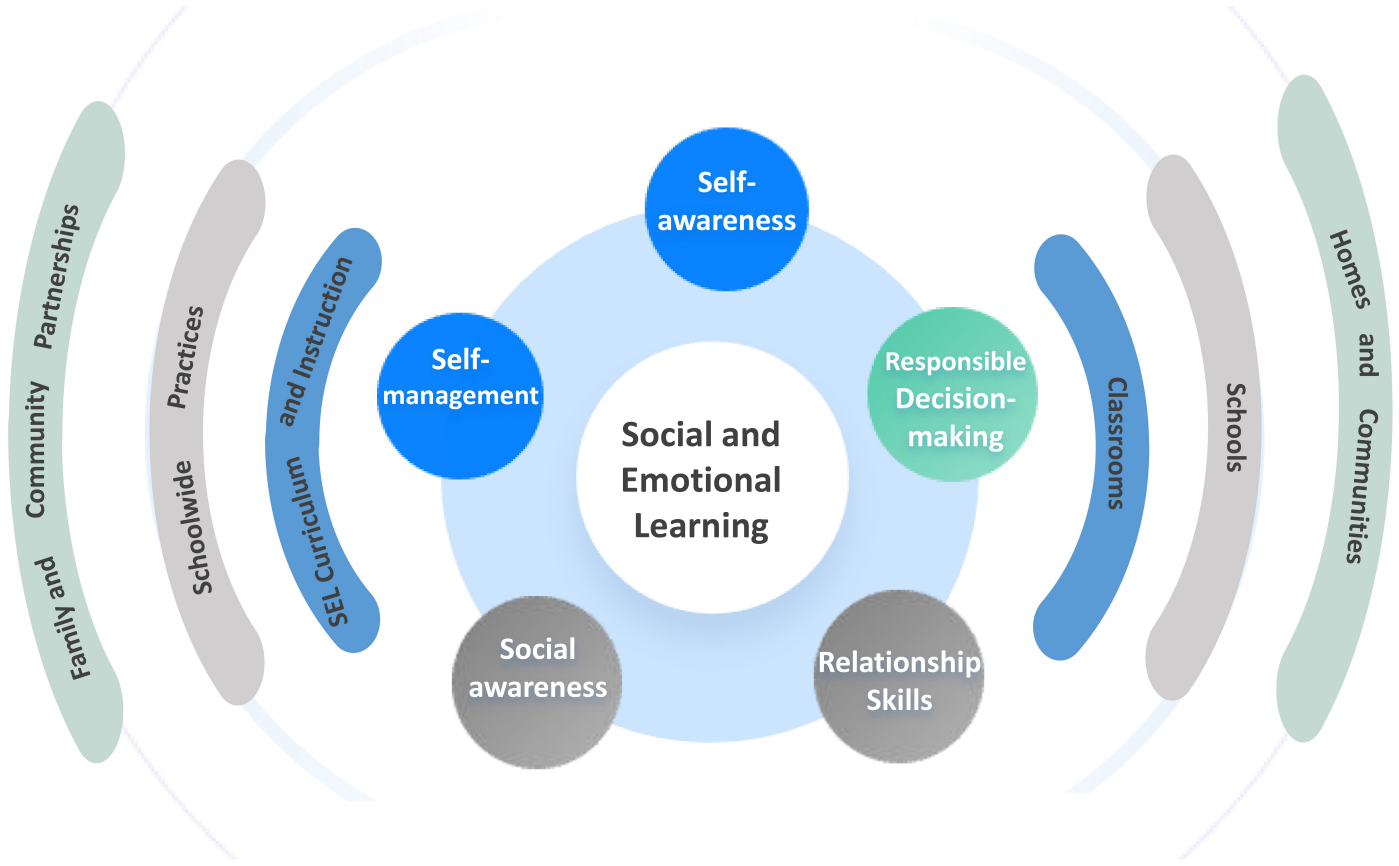
The CASEL 5 framework for social and emotional learning (SEL). The figure illustrates the five core competencies of SEL and the key settings for their implementation, as conceptualized by the Collaborative for Academic, Social, and Emotional Learning. Source: Adapted from the Collaborative for Academic, Social, and Emotional Learning (https://casel.org/). Visualization by the authors.

**Operational framework and core dimensions:** The framework organizes SEC into five broad, inter-related competency domains (Figure 2): (i) self-awareness, an ability to precisely evaluate one's emotions, interests, values, strengths, and talents, as well as sustain a fundamental sense of self-confidence; (ii) self-management, an ability to control emotions, manage stress, overcome impediments, set goals, and express emotions constructively; (iii) social awareness, an ability to identify and appreciate similarities among people and groups from others' viewpoints, follow social behavioral norms, and recognize and use resources from family, school, and the community; (iv) relationship skills, an aptitude to cultivate and sustain healthy and beneficial relationships, resist social pressure, avert and resolve interpersonal conflicts, and seek assistance when needed; (v) responsible decision-making, an ability to make decisions based on ethical standards, safety deliberations, apt behavioral norms, and respect for others, while also envisaging potential consequences and applying decision-making skills to learning and living environments (McKown, 2019).

**Measurement and cultural adaptation:** There is no single “CASEL test.” Instead, SEC measurement relies on a diverse array of psychometrically sound tools, such as the Devereux student strengths assessment (DESSA) (Anthony et al., 2022) and the social skills improvement system–social–emotional learning edition (SSIS SEL) (LeBuffe et al., 2018), which are aligned with the CASEL framework. Measurement relies on a multitude of aligned tools (e.g., DESSA, SSIS SEL). The complexity of SEL assessment is compounded when applied in cross-cultural contexts, necessitating rigorous cultural adaptation and contextualization of both the assessment tools and the intervention programs themselves (Abd Hadi et al., 2023; Al-Hroub and Al-Hroub, 2024; Johnson and Whitcomb, 2025). This adaptation extends beyond mere linguistic translation to ensure conceptual, linguistic, and psychometric equivalence across cultures (Cubaka et al., 2018; Khan et al., 2022; Mirza et al., 2022). For instance, a direct translation of an instrument developed in one culture may lose its content validity when applied in another, as idioms or specific behaviors may not carry the same meaning or relevance (Johnson and Whitcomb, 2025). The Manchester translation evaluation checklist (MTEC) is one such tool designed to assess the quality of translation and cultural adaptation of cognitive tests, highlighting the need for systematic approaches (Mirza et al., 2022). Cultural adaptation involves several critical steps, including cognitive interviews, expert review, pilot testing, and validation within local educational and developmental contexts (McKown, 2019; Mirza et al., 2022; Abd Hadi et al., 2023). This process aims to ensure that the assessed competencies are meaningful and reflect local values and educational ethos (McKown, 2019; Abd Hadi et al., 2023). Research highlights the need for a “bottom-up” approach in developing culturally relevant SEL frameworks and measurement tools, moving away from top-down development that may lead to confirmation bias (Anziom et al., 2021; Vinal and Renshaw, 2024). For example, a study in Cameroon developed an SEL framework and measurement tool through participatory and interactive methods with indigenous Baka communities, ensuring context specificity (Anziom et al., 2021). Similarly, Malaysian researchers explored parents' and teachers' cultural conceptualizations of adolescent social and emotional competencies to inform culturally sensitive SEL interventions (Abd Hadi et al., 2023).

**Critiques and cultural considerations:** Critics emphasize that effective adaptation requires significant local expertise to ensure that the competencies are culturally appropriate and meaningful (Anziom et al., 2021; Abd Hadi et al., 2023). This is particularly important because the interpretation of behaviors and the expression of emotions can vary significantly across cultures (Lee, 2023). For instance, secure attachment patterns may have health-protective effects among African American girls in psychiatric care, highlighting the cultural nuances in social–emotional development (Emerson et al., 2012). Studies have also addressed the cultural relevance and social validity of the SSIS SEL Classwide Intervention Program, incorporating insights from teachers in authentic settings (Hart et al., 2023).

#### 3.2.2 A comparative analysis of major social and emotional competence frameworks

The OECD's approach represents a concerted effort to establish a universal, empirically grounded metric for SEC, primarily for large-scale international comparison and policy benchmarking.

**Theoretical foundation:** The framework is explicitly anchored in the five-factor model (Big Five) of personality, chosen for its robust empirical evidence, hierarchical structure, and demonstrated cross-cultural applicability and predictive validity for life outcomes. This represents a personality-trait-based, individual-differences perspective on SEC (Chernyshenko et al., 2018).

**Figure 3:**
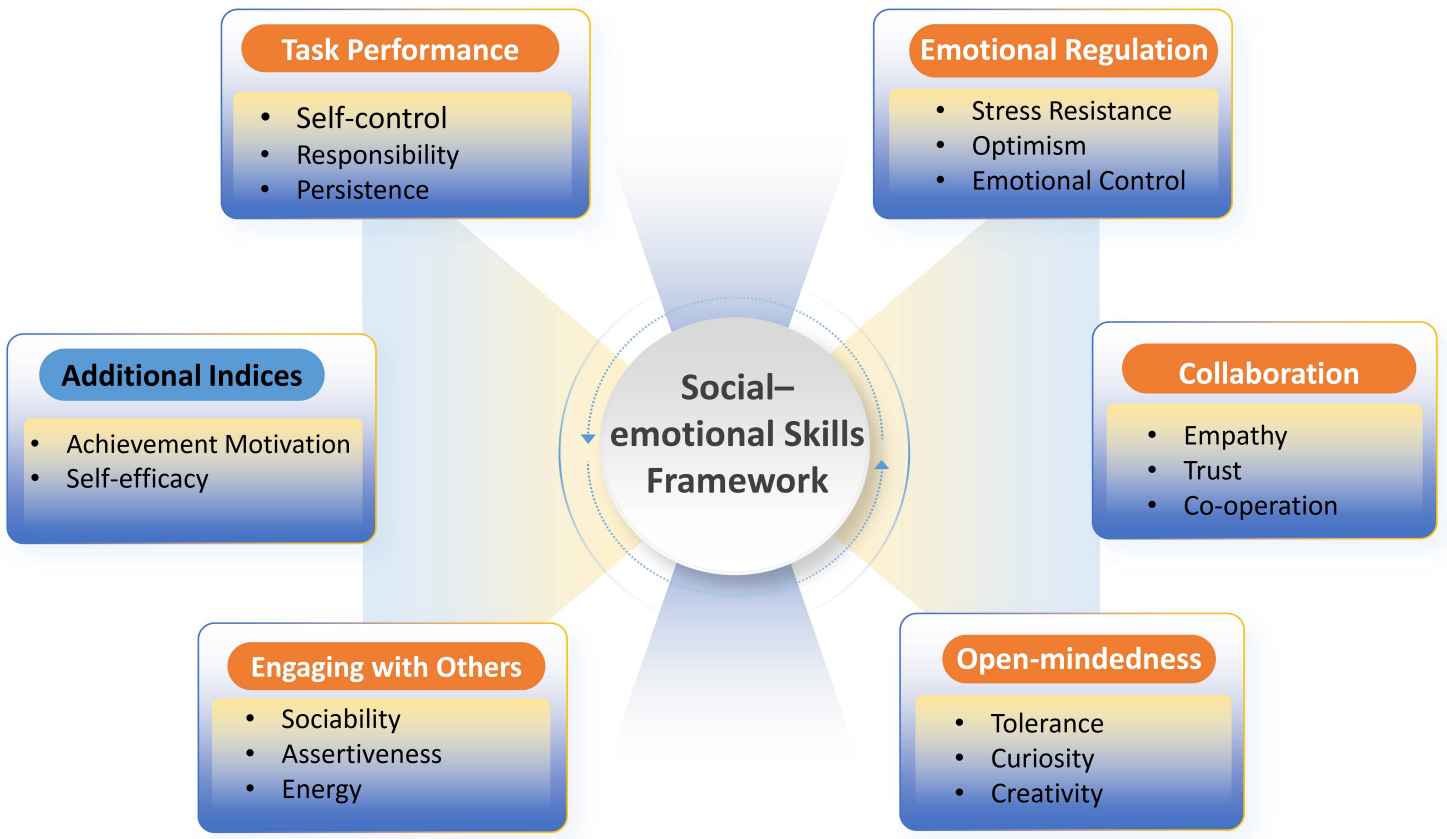
The conceptual framework of the OECD Study on Social and Emotional Skills (SSES). The framework illustrates the mapping of specific, learnable social and emotional skills onto the broader “Big Five” personality domains, forming the conceptual basis for the OECD Study on Social and Emotional Skills (SSES). Source: Adapted from Kankaraš and Suarez-Alvarez (2019). Visualization by the authors.

**Operational framework and core dimensions:** The Big Five domains are directly mapped onto a set of 15–17 specific, learnable “social and emotional skills” (see Figure 3): (i) *conscientiousness*, depicts diligence and goal-oriented behavior; (ii) *emotional stability*, an ability to manage negative emotional experiences and stress, which is vital in emotional regulation; (iii) *agreeableness*, reflects traits like caring for others, humility, and trustworthiness; (iv) *openness*, covers curiosity, creativity, and inclusivity; (v) *extraversion*, portrays energy, positive emotions, and confidence. The Big Five personality model offers a universal, empirically grounded framework for unifying otherwise disordered and independent social–emotional elements (Walton et al., 2023). Besides, the personality traits in the Big Five personality model are measurable, and extensive empirical evidence connects these traits to educational success, wellbeing, health, and job performance (Chernyshenko et al., 2018).

**Measurement and cross-cultural validation:** The SSES framework, which includes a short form (SSES-SF) with 18 items across five broad skill domains, aims to provide policy makers and educators with tools to foster social and emotional learning (Wang and King, 2024; Walton et al., 2023). A significant challenge in international surveys like the SSES is ensuring cross-cultural validity and comparability of results (Avvisati et al., 2019). To address this, the SSES employs a rigorous cross-cultural adaptation protocol involving extensive translation, cognitive pretesting, and the use of anchoring vignettes to adjust for differential item functioning (DIF) across participating cities (Avvisati et al., 2019; Paccagnella, 2021). Anchoring vignettes are a powerful instrument used to detect systematic differences in the use of self-reported ordinal survey responses, helping to mitigate bias caused by varying response styles across different populations (Paccagnella, 2021; Menold et al., 2023; Runge and Soellner, 2024). Without such methods, variations in how individuals interpret and use response categories can lead to biased measurements and invalidate cross-cultural comparisons (Paccagnella, 2021). For instance, recent research highlights the utility of anchoring vignettes in adjusting for bias in parent-reported mental health measures across diverse countries (Runge and Soellner, 2024) and in ensuring data comparability in heterogeneous refugee samples (Menold et al., 2023). The goal is to achieve scalar measurement invariance, which ensures that the questions are understood and answered in the same way in different cultural contexts, thereby enabling valid score comparisons (Avvisati et al., 2019; Menezes Cunha et al., 2021; Menold et al., 2023). Configural, metric, and scalar measurement invariance are indicators of bias-free statistical cross-group comparisons, though they are often difficult to verify (Menold et al., 2023). Measurement invariance ensures that a test measures the same underlying concept in the same way across different groups, with scalar invariance being essential for validly comparing average scores of a latent trait between groups. Failure to establish invariance can lead to scientifically invalid conclusions where observed differences are artifacts of measurement bias.

**Critiques and cultural considerations:** The SSES framework has faced critiques regarding its underlying philosophical and cultural assumptions. The framework is often perceived as individualistic and economically oriented, potentially prioritizing skills that enhance productivity and adaptability within market economies (Williamson, 2019). Critics argue that this emphasis may undervalue communal, relational, or spiritual dimensions of wellbeing that are prominent in collectivistic cultures (You, 2023). For example, the concept of social–emotional learning, while originating in North America, needs to consider diverse cultural perspectives such as those rooted in Confucianism and Daoism to be truly globally applicable (You, 2023). This points to a broader concern about imposed etics in cross-cultural research, where instruments developed in one culture are applied to another without establishing local meaning or equivalence, potentially leading to invalid conclusions. Furthermore, some interpretations of the OECD report stress the nurturing of extraversion, which suggests a cultural bias toward extroverted traits over introverted ones (Cefai et al., 2018; Blevins et al., 2021). The Big Five personality model includes extraversion as a core dimension, characterized by sociability, assertiveness, and energetic behavior (Kang et al., 2024). While extraversion is often linked to success in various life domains, an overemphasis can lead to the marginalization or misinterpretation of introverted traits (Blevins et al., 2021). Introversion, characterized by a preference for less external stimulation and more reflective thought, is not a deficit but an adaptive and contextually meaningful expression of SEC (Blevins et al., 2021). Studies have shown the importance of Big Five personality traits in predicting successful transitions from school to vocational education (Nießen et al., 2020) and their relationship with social entrepreneurship intentions (Luc, 2022). However, the differential impact of introverted traits within this framework requires further investigation to ensure a balanced value judgment of introverted and extroverted personality traits for individual progress (Cieciuch and Strus, 2021).

#### 3.2.3 Evolving conceptualizations in the Chinese context

Research in China reveals a dynamic landscape where global SEC concepts interact with deep-seated Confucian, collectivist, and socialist values, leading to distinct emphases and hybrid models (You, 2023).

**Theoretical foundation:** The theoretical foundation of SEC in the Chinese context is strongly influenced by Confucian ethics, which emphasize social harmony, filial piety (xiao), relational hierarchy, and the cultivation of virtue (de) (Du and Li, 2024). Unlike Western models that often prioritize an individualistic, intrapsychic focus, Chinese conceptualizations of SEC view it as integral to moral education and the development of a “whole person” who contributes to societal good (Du and Li, 2024). For instance, concepts like *Ren* (benevolence), Li (ritual propriety), *Yi* (righteousness), *Xiao* (filial piety), and *Zhi* (wisdom) are integrated into educational curricula to shape both moral and intellectual development (Du and Li, 2024). These traditional values underscore the importance of relationship-oriented thinking and collective wellbeing (Li et al., 2024). The Confucian perspective on the self is not isolated and static but is understood as a center of relationships engaging in a continuous process of moral cultivation and transformation through constant interaction.

**Operational framework:** Chinese scholars and policymakers adapt international SEC frameworks by re-prioritizing dimensions, placing greater emphasis on competencies that foster group harmony, respect for authority, perseverance, and collective responsibility (McCabe et al., 2023; You, 2023). For example, while “self-management” in Western contexts often emphasizes personal autonomy and goal-setting, in the Chinese context, it may be framed more as self-discipline for the sake of group goals (You, 2023). This is rooted in a collectivist culture where individual identity is intertwined with group identity (McCabe et al., 2023). Similarly, “social awareness” is frequently interpreted as “perspective-taking and empathy for others” and “respect for elders and authority,” reflecting the hierarchical and relational nature of Chinese society (Xu et al., 2025). These adaptations are not merely translations but rather epistemologically distinct reconstructions informed by millennia-old pedagogical traditions and contemporary national education policies (You, 2023). The concept of *qing shang* (emotional intelligence) has been widely adopted in China but is often interpreted through a relational lens (Chuan Chu, 2018). *Qing*, often translated as “emotions,” differs from the contemporary Western notion in that its scope includes likes, dislikes, and desires, and it frequently refers to the condition of the heart/mind underlying human responses (Shun, 2022). Emotional intelligence, therefore, often emphasizes emotional attunement to others' needs and situational appropriateness (yi) rather than purely internal emotional clarity or expression (Chuan Chu, 2018). This relational interpretation aligns with the overarching goal of social harmony, where appropriate emotional expression and management contribute to stable interpersonal relationships and collective wellbeing.

**Measurement and cultural adaptation:** The development of culturally resonant measurement tools in China involves a dual-track approach: the rigorous transcultural adaptation of established Western instruments and the indigenous development of new scales. This process extends beyond simple translation to include meticulous back-translation, cognitive interviewing, and extensive psychometric validation within Chinese populations. Instruments like the Chinese versions of the social emotional competence questionnaire (C-SECQ) and the social–emotional learning scale (C-SELS) exemplify this effort (Tan et al., 2025). They have demonstrated robust reliability and construct validity in local samples, yet factor analyses frequently reveal culturally distinct constructs or altered factor loadings compared to their Western origins. A recurring and significant finding is the emergence and salience of dimensions centered on “Relationship with Parents” or “Family Orientation,” reflecting the foundational role of *xiao* (filial piety) and intergenerational dynamics in Chinese SEC (Zong et al., 2024). This contrasts with Western tools where such familial focus is often less explicit or subsumed under broader relationship categories. Empirical research, such as studies on early childhood educators' perceptions, further operationalizes how core competencies like “social awareness” are interpreted to include specific respect for elders and authority, guiding item development and validation (You, 2023).

**Critiques and cultural considerations:** The rapid importation and adaptation of Western SEC models have sparked vigorous scholarly debate regarding cultural appropriateness and epistemic sovereignty. Critics caution against “psychological imperialism,” arguing that relying solely on adapted frameworks risks imposing foreign constructs that may neglect or distort locally meaningful aspects of SEC, such as the relational interpretation of *qing shang* (emotional intelligence) (Chuan Chu, 2018). This has amplified calls for genuine indigenous theory-building grounded in Confucian, Daoist, and other Chinese philosophical traditions. A central, ongoing tension lies in balancing the cultivation of individual creativity and self-expression—increasingly valued in 21st-century educational and economic discourse—with the deep-seated traditional values of social harmony, conformity, and collective responsibility (You, 2023). Furthermore, some critiques highlight the risk that certain interpretations of collectivist and hierarchical values, if not carefully implemented, could be misused to justify excessive social control or oppressive norms, as seen in discussions linking distorted Confucian ethics to harsh work cultures. The future trajectory points toward the development of synthesized frameworks that neither simply westernize nor essentialize “Chineseness,” but rather thoughtfully integrate traditional virtues with modern psychological constructs to support holistic, contextually-grounded development.

#### 3.2.4 The Australian (and Aotearoa New Zealand) frameworks: integrating wellbeing and indigenous perspectives

The integration of socio-emotional competence (SEC) with broader wellbeing frameworks, particularly through the inclusion of Indigenous perspectives, is exemplified by models developed in Australia and Aotearoa New Zealand. These frameworks move beyond universalistic conceptualizations of wellbeing to embrace contextualized, ecological, and spiritually-attuned foundations (Sangha et al., 2024).

**Theoretical foundation:** These frameworks often blend SEC with positive psychology, resilience theory, and holistic models of child wellbeing. Crucially, they are increasingly informed by Indigenous knowledge systems, such as Aboriginal and Torres Strait Islander perspectives in Australia (Dudgeon et al., 2025) and Māori concepts like te whare tapa whā (the four-sided house model of health) in Aotearoa New Zealand (Mooney et al., 2020). This represents a shift toward more contextualized, ecological, and spiritually-attuned foundations.

**Operational framework:** In Australia, wellbeing frameworks are increasingly incorporating Aboriginal and Torres Strait Islander perspectives, recognizing that wellbeing is deeply intertwined with connection to Country, kinship, and intergenerational knowledge (Dudgeon et al., 2025). This approach is crucial given the disproportionate representation of Aboriginal children in out-of-home care and the need for culturally situated, trauma-informed care delivered by Aboriginal practitioners (Lukey et al., 2023). In Aotearoa New Zealand, the Māori wellbeing model of Te Whare Tapa Whā conceptualizes health and wellbeing across four interconnected dimensions: taha wairua (spiritual health), taha hinengaro (mental health), taha tinana (physical health), and taha whānau (family health) (Purdy, 2020). Spirituality, taha wairua, is considered an inseparable aspect of holistic wellness within this framework.

**Measurement and cultural considerations:** The development of culturally safe and responsive assessment practices is central to Australian and New Zealand frameworks. This necessitates a fundamental methodological shift: from standardized, decontextualized testing toward relationship-centered, community-based approaches (Strauss-Hughes et al., 2022). It involves participatory design, empowering Indigenous communities to lead in tool development and interpretation. Assessments thus embrace a wider range of methodologies, moving from purely quantitative measures to incorporate narrative evaluations, conversation-based observations, and the monitoring of wellbeing indicators defined by the communities themselves. The “Wellah Together Online” initiative serves as a practical example of this diversified, community-led approach to assessment (Mulder et al., 2023). The core lies in adopting methodologies that honor Indigenous ways of knowing centered on “place, story, and relationship”—for instance, using in-depth dialogues rather than surveys to understand the impact of connection to country on wellbeing.

**Critiques and cultural considerations:** The core challenge is achieving meaningful integration, not the superficial grafting of Indigenous concepts onto existing Western SEC structures (Lee and Junus, 2024; Hoosen et al., 2024). This requires vigilance against the risk of tokenistic inclusion—merely listing concepts like “connection to land” as a dimension while measuring it with individualized, decontextualized scales. True integration demands a paradigm shift that acknowledges holistic worldviews, such as the Māori model Te Whare Tapa Whā, which views spiritual, family, mental, and physical health as an indivisible whole. This means it is essential to decentralize Western models and create space for equally valid conceptualizations of SEC and wellbeing rooted in Indigenous knowledge systems—conceptualizations that acknowledge historical trauma and collective wellness.

## 4 Discussion

### 4.1 Synthesis of cross-cultural divergence and convergence

This review elucidates a spectrum of approaches to SEC, reflecting fundamental cultural variations in the understanding of the self, relationships, and the “good life.” The OECD framework exemplifies a universalist, metric-driven approach, seeking a common currency for global comparison rooted in personality psychology. In contrast, the CASEL framework is a pragmatist, intervention-oriented model designed for educational systems, whose transportability depends on deep cultural adaptation. The Australian/Aotearoa New Zealand examples illustrate a contextualist, integrative turn, weaving SEC into broader wellbeing agendas and engaging with indigenous paradigms. The Chinese context showcases a dialectical process of adaptation, where global constructs are filtered and reshaped through the lens of local philosophical and social values.

Despite differences, convergence exists around the recognition of core human capacities related to emotion, relationships, and goal pursuit. However, the priority, manifestation, and desired endpoints of these capacities differ markedly (Lee and Junus, 2024). A collectivistic lens tends to foreground harmony, duty, and context-sensitive emotional regulation, while an individualistic lens may foreground autonomy, self-expression, and self-contained emotional clarity.

### 4.2 The pivotal challenge of measurement invariance

A central methodological and ethical challenge in cross-cultural SEC research lies in establishing measurement invariance—the statistical property confirming that a construct is measured equivalently across groups. Without invariance, observed differences may reflect measurement artifacts rather than true cultural variations. The hierarchical “ladder of invariance” outlines progressive requirements: configural invariance (same factor structure), metric invariance (equal factor loadings, enabling comparison of relationships), and the most stringent scalar invariance (equal item intercepts, enabling direct mean score comparison) (Żemojtel-Piotrowska et al., 2018). Achieving full scalar invariance for SEC constructs is notoriously difficult, as items and response scales are interpreted through distinct cultural filters regarding self-presentation, emotional expression, and social norms (Thomas et al., 2023). For instance, concepts like “assertiveness” or “happiness” may carry culturally specific meanings and desirability.

Techniques like anchoring vignettes (used by the OECD) are employed to calibrate differing response styles by providing common reference points. However, they are a partial solution that addresses scale interpretation more than fundamental construct equivalence. The pursuit of “perfect” invariance for cross-cultural comparison must be balanced against the risk of forcing culturally distinct phenomena into a single, potentially reductionist, measurement model, a form of methodological imperialism (Menold et al., 2023). Therefore, the field benefits from a pluralistic measurement strategy. This approach combines carefully adapted standardized tools, rigorously tested for invariance, with culturally situated qualitative assessments. It advocates for an emic–etic integration, where local, culturally-grounded understandings of SEC (emic) inform the development and evaluation of tools intended for cross-cultural use (etic), thereby moving beyond the uncritical imposition of externally derived instruments.

### 4.3 Technological potential: toward multimodal and culturally contextualized assessment

Research indicates that artificial intelligence (AI) holds significant potential to simulate and enhance SEC, particularly in education and mental health support (Indellicato, 2024). Its integration represents a transformative frontier for cross-cultural assessment, offering unprecedented potential to transcend the limitations of traditional self-report measures by enabling more ecologically valid, behaviorally anchored, and multifaceted evaluations. However, a crucial caveat is that AI cannot replicate genuine human empathy and emotional experience (Perry, 2023). Therefore, the primary objective for future research and application must be to strategically leverage AI's analytical and scalable strengths—such as its capacity for personalized feedback, immersive simulation, and complex pattern recognition—to augment human social–emotional skills, not to replace them (Indellicato, 2024). Realizing this goal in a culturally responsive and equitable manner necessitates proactively managing its profound risks, including algorithmic bias, the “black box” problem threatening cultural validity, and issues of data sovereignty (Zhao et al., 2025). The ultimate imperative is to ensure these powerful technologies are developed and deployed to genuinely serve human wellbeing and sustainable societal development.

AI and associated digital tools are catalyzing a paradigm shift in how SEC can be operationalized and measured, moving beyond static questionnaires toward dynamic, context-rich assessment.

**Generative AI for assessment material creation:** AI is revolutionizing the very *development* of assessment tools. Generative AI models can automatically create culturally varied and controlled stimuli for SEC tests. This capability is underpinned by AI's growing proficiency in understanding cross-cultural emotional expression. For instance, deep learning models have been successfully applied to analyze a large-scale, multicultural dataset comprising hundreds of thousands of facial expressions generated by participants from diverse countries, with interpretations validated in their native languages (Brooks et al., 2024). This demonstrates AI's capacity to process and interpret nuanced cross-cultural emotional data. Leveraging this understanding, generative AI can synthesize culturally informed, high-fidelity facial expressions or social scenarios for emotion perception and social cognition tests, dramatically improving the scalability and cultural adaptability of assessment development.

**Multimodal behavioral analysis:** The fusion of computer vision, vocal analytics, and sensor data allows for the objective, continuous measurement of behavioral correlates of SEC. AI algorithms can analyze facial micro-expressions, prosody in speech, gesture, and physiological signals (e.g., from wearables) as individuals interact with simulated or real-world tasks (Licht, 2025). This multimodal approach captures the implicit, non-verbal dimensions of SEC that are crucial for cross-cultural understanding but often elusive in self-report.

**Natural language processing (NLP) for social–emotional discourse:** Advanced NLP and large language models (LLMs) enable deep analysis of written or spoken language to assess emotional vocabulary, empathy, perspective-taking, and collaborative communication styles (Ghafoor et al., 2025). This can be applied to analyze responses to open-ended scenarios, group discussions, or digital communication traces, providing insight into culturally nuanced social–emotional reasoning and expression.

**Immersive simulation with virtual/augmented reality (VR/AR):** VR/AR technologies can place individuals within standardized, yet culturally configurable, social simulations. Researchers can observe and code real-time behavioral responses—such as conflict resolution, cooperation, or emotional regulation—within these safe, repeatable, but ecologically rich environments. This approach allows for the assessment of SEC as it unfolds in action within tailored cultural contexts. For example, a participant's ability to navigate a collaborative task within a VR simulation of a multicultural team meeting can be assessed by analyzing not only their decisions and verbal responses, but also their non-verbal cues, such as eye contact, gesture synchrony, and vocal tone, which are captured and processed by integrated multimodal sensors and AI (Nyaaba et al., 2024).

**Predictive modeling and automated profiling:** Machine learning models can be trained on large-scale, multimodal datasets to identify complex behavioral patterns predictive of specific SEC dimensions (e.g., self-regulation, social awareness), thereby serving as efficient preliminary profiling tools. For example, research utilizing multimodal data—such as video and electro dermal activity—has successfully identified key behavioral patterns and pivotal moments within collaborative interactions (Järvelä et al., 2023). This demonstrates machine learning's capacity to parse intricate social–behavioral signals and generate deeper insights. Furthermore, the emerging capability of LLMs to simulate psychological constructs and cultural variables provides a novel “sandbox” environment for theoretically exploring the cross-cultural manifestations of SEC.

## 5 Conclusion

This systematic review demonstrates that social and emotional competence is a quintessentially cultural construct. The dominant frameworks analyzed—OECD, CASEL, Australian, and the evolving Chinese models—are not neutral scientific discoveries but cultural artifacts, reflecting specific values, histories, and goals. The OECD seeks a universal metric for global policy; CASEL provides a flexible toolkit for educational reform; Australian frameworks attempt synthesis with indigenous worldviews; and Chinese scholarship navigates the integration of global science with local virtue ethics.

The future of the field lies in moving beyond the unilateral adaptation of Western models and embracing a genuinely pluralistic, dialogical approach. This involves recognizing multiple valid epistemologies of SEC, investing in indigenous theory-building, and developing assessment methodologies that balance the need for generalizable knowledge with deep cultural respect. Technology, if guided by strong ethical principles of equity and co-design, can aid this process by providing new, context-rich methods for understanding development. Ultimately, enhancing the social–emotional wellbeing of the world's youth requires not a single, perfect framework, but the wisdom to nurture diverse competencies that enable young people to thrive within, and positively transform, their own unique cultural worlds.

## Data Availability

The raw data supporting the conclusions of this article will be made available by the authors, without undue reservation.
